# Genome-Wide Association Studies Identifies Seven Major Regions Responsible for Iron Deficiency Chlorosis in Soybean (*Glycine max*)

**DOI:** 10.1371/journal.pone.0107469

**Published:** 2014-09-16

**Authors:** Sujan Mamidi, Rian K. Lee, Jay R. Goos, Phillip E. McClean

**Affiliations:** 1 Genomics and Bioinformatics Program, North Dakota State University, Fargo, North Dakota, United States of America; 2 Department of Plant Sciences, North Dakota State University, Fargo, North Dakota, United States of America; 3 Department of Soil Science, North Dakota State University, Fargo, North Dakota, United States of America; National Institute of Plant Genome Research (NIPGR), India

## Abstract

Iron deficiency chlorosis (IDC) is a yield limiting problem in soybean (*Glycine max* (L.) Merr) production regions with calcareous soils. Genome-wide association study (GWAS) was performed using a high density SNP map to discover significant markers, QTL and candidate genes associated with IDC trait variation. A stepwise regression model included eight markers after considering LD between markers, and identified seven major effect QTL on seven chromosomes. Twelve candidate genes known to be associated with iron metabolism mapped near these QTL supporting the polygenic nature of IDC. A non-synonymous substitution with the highest significance in a major QTL region suggests soybean orthologs of *FRE1* on Gm03 is a major gene responsible for trait variation. NAS3, a gene that encodes the enzyme nicotianamine synthase which synthesizes the iron chelator nicotianamine also maps to the same QTL region. Disease resistant genes also map to the major QTL, supporting the hypothesis that pathogens compete with the plant for Fe and increase iron deficiency. The markers and the allelic combinations identified here can be further used for marker assisted selection.

## Introduction

Iron (Fe) is an essential element for multiple plant functions including photosynthesis, respiration, chlorophyll biosynthesis, and redox reactions in plants, and is a structural component in heme, the Fe-sulfur cluster, and Fe-binding sites. To avoid toxicity, plants control the uptake, utilization, and storage in response to Fe availability [1]. Despite its abundance in the soil, Fe is only slightly soluble under aerobic conditions, especially in high-pH and calcareous soils [2]. Under these conditions, iron deficiency leads to developmental defects, including chlorosis, growth retardation, and reduced crop productivity [3]. Iron deficiency chlorosis (IDC) is an important yield-limiting factor for soybeans (*G. max*) grown on calcareous soils that have a high percentage of calcium carbonate and soluble salts. This soil type is common in the north-central regions of the United States [4] where soybean is widely grown. In these soils, interactions of high pH, carbonate and high field moisture content at planting leads to early IDC symptoms that effect yield. It is estimated that the current revenue losses due to IDC in soybean are $260 million [5].

IDC results from the inability of susceptible genotypes to efficiently mobilize iron into the plant when it is growing in high pH calcareous soils. In these soils, ferrous iron is not readily released from soil particles, and subsequently iron availability is limited. Based on its response to Fe availability, soybean is considered a Strategy I plant [6] that reduces ferric chelates at the root surface and absorbs ferrous ions across the plasma membrane of the root [2]. Other processes involved in Strategy I include excretion of proton and phenolic compounds from the roots to the rhizosphere, which increases the solubility of ferric ions and support the reducing capacity of ferric Fe on the root surface [2]. Strategy I plants also increase root hair formation, thereby increasing the surface area available for iron uptake [7]. Once iron enters the root, membrane transporters move Fe into the xylem where it chelates with citrate. The chelated form of iron then moves through the xylem stream to growing leaves. Iron is then remobilized from the leaves, forms a complex with nicotianamine, and is transported via the phloem to younger leaves and seeds. Excess water leads to an elevated concentration of bicarbonates in the root apoplast that impedes the Fe^3+^– chelate reductase activity necessary for the conversion of Fe^3+^ to Fe^2+^. Bicarbonates also immobilize the movement of iron to young leaves once it is absorbed at the root level [8].

Given the many physiological mechanisms associated with iron metabolism, it is not surprising that IDC is a complex quantitative trait with multiple genetic factors [9]. In addition to the genes involved in Fe acquisition, translocation and compartmentalization within the organelles, there are other genes involved in Fe transcriptional and post transcriptional changes that are expressed/repressed under deficient and excess conditions or affect iron homeostasis pathways [10]–[17].

Genetic experiments using bi-parental mapping populations have identified multiple quantitative trait loci (QTL) associated with IDC in soybean [11]–[12]. O’Rourke et al. [13] identified several genes induced under iron-limiting conditions using a microarray study. Severin et al. [14] discovered multiple introgressed genomic regions responsible for IDC in near isogenic lines, and Stec et al. [15] further refined these introgression regions. Recently, Peiffer et al. [5] evaluated candidate genes on Gm03.

A genome-wide association study (GWAS) is an excellent approach to discover genetic factors in a population because of the high number of recombinant events the population represents. GWAS uses the linkage disequilibrium (LD) pattern in a large population of unrelated individuals [16] to map significant effect loci. Since IDC is a complex trait, utilizing GWAS can identify major factors controlling the IDC response in soybean. Previously, Wang et al. [17] used GWAS to identify simple sequence repeat (SSR) markers associated with IDC in two independent populations. Later, nine shared SNP were discovered and confirmed by Mamidi et al. [9] in two separate populations. The objective of this study was to identify QTL and genes involved in IDC of soybean using a GWAS population and a new high density SNP map obtained through genotype-by-sequencing (GBS). Those results were then translated into SNP markers that can be used for marker assisted selection (MAS).

## Materials and Methods

### Phenotype and genotyping

Two plant populations consisting of unique set of advanced soybean breeding lines developed for the north-central region of the US were grown in years 2005 (n = 132) and 2006 (n = 138). IDC screening protocols and DNA isolation procedures were described previously [9]. The trait was rated on a scale of 1 to 5, where 1 indicates no chlorosis and a normal green plant, 2 indicates slight yellowing of upper leaves and the leaf veins, 3 indicates interveinal chlorosis in the upper leaves with no stunning of growth or death of tissue (necrosis), 4 indicates interveinal chlorosis of the upper leaves along with some apparent stunning of growth or necrosis of tissue, and 5 indicates severe chlorosis plus stunned growth and necrosis in the youngest leaves and growing points [9]. Phenotypic data was adjusted based on least squares means for each population independently.

GBS libraries were prepared and analyzed at the Institute for Genomic Diversity (IGD), Cornell University, according to Elshire et al. [18], using the enzyme *Ape*KI. The GBS analysis pipeline [3.0.128], an extension of the Java program TASSEL [19], was used to call SNPs from the sequenced GBS library. Reads were aligned to the soybean reference genome v1.1 [20] using BWA 0.6.1 [21]. VCFtools (v0.1.8) [22] was used to summarize the SNP data.

The data was analyzed by combining the two populations (which are of similar size) to account for unique alleles that are at a low frequency in one or both populations. These low frequency alleles can have a significant phenotypic effect in a small set of genotypes, but that effect cannot be detected in a normal statistical framework [16].

### Genome-wide association study

#### Imputation

fastPHASE 1.3 [23], a likelihood-based imputation software, was used with default settings to impute missing data for the combined population. Imputation analysis was performed to increase the power of the study and fine map the causal variant [24]. A minor allele frequency (MAF) of 0.05 was used as cutoff, since variants at low frequency have little power to detect association with the phenotype [16].

#### Linkage disequilibrium (LD)

The LD between markers was estimated as the partial squared allele frequency correlation (r^2^) using CORR procedure in SAS 9.3. Three principal components (PC) that explain about 25% of cumulative variation were used as cofactors. We used a partial correlation because unlinked loci can be in LD simply because of population structure [25]–[26]. The decay of r^2^ with physical and/or genetic distance between loci is often used to determine the density of markers to use in whole genome association scans [27] whereas local LD on chromosomes is used to account for genes/QTL associated with trait variation. Overestimation of LD can lead to misinterpretations either on the extent of LD decay or on the size of the QTL [26]. LD decay graphs were plotted with physical distance (Mbp) vs. r^2^ for all intra-chromosomal comparisons using nonlinear regression as described by Remington et al. [28]. The expected decay of LD was estimated as described by Pyhajarvi et al. [29]. We fit this equation into a nonlinear regression model using NLIN procedure in SAS 9.3.

#### Population structure, kinship, and model testing

A principal component analysis was utilized to control for population structure [30]. PCs were estimated using the PRINCOMP procedure in SAS 9.3. The PCs that explained 25% (PC_25_) and 50% (PC_50_) cumulative variation were selected for the analysis. In addition an identity-by-state matrix [31] was used to control for relatedness estimated as a centered relatedness matrix in Gemma 0.92 [32]. Gower’s similarity coefficient was calculated using the DISTANCE procedure in SAS 9.3 to measure the relatedness by state of the individuals.

To test for marker-trait associations, the MIXED procedure in SAS for the three linear models that do not have kinship (Naïve, PC_25_ and PC_50_) was used. Since approximate tests compromise the analysis of complex genetic architectures when one genetic factor masks another [33], we used the exact test implemented in Gemma 0.92 to estimate marker significance [32] for the other three models with kinship (Kinship, PC_25_+kinship, PC_50_+kinship). As the standard errors follow a uniform distribution [34], we used a rank based mean squared difference (MSD) for model selection as described in Mamidi et al [9].

#### Marker-trait associations

Markers are defined as significant at two levels, within 0.01 percentile, and 0.1 percentile tails of the empirical p-value distribution of 10,000 bootstraps. This method of using an empirical distribution of data as a replacement for a population with an unknown distribution has the advantage of simplicity and provides an efficient and precise estimation [35]–[36]. This approach is similar to choosing an arbitrary value but instead it is based on choosing a predefined percentile tail from an empirical distribution. Here, the confidence intervals for an empirical distribution were obtained from 10,000 bootstraps over ∼35,000 markers distributed throughout the genome. We chose this approach over a cutoff because p-value is dependent on the distribution of phenotype, the variation explained by the marker, structure and relatedness of the population, and heritability of the trait.

The means for the alleles were calculated using MEANS procedure in SAS. The percent of variation explained by the marker is calculated using a simple regression (GLM procedure) in SAS. The additive effect of the variant allele is calculated as half the difference between IDC mean of the variant allele and IDC mean of the reference allele.

To determine the minimal number of SNPs independently associated with IDC, stepwise regression analysis was applied to all significant markers from a single-SNP analysis at a significance level of 0.1 percentile [37]. In addition, a multiple R^2^ value for all these loci was also estimated [9]. A significant p-value of 0.05 was necessary for both marker and model for stepwise inclusion of the marker in REG procedure in SAS 9.3.

#### Annotation of SNP

To characterize SNPs as intergenic, UTR, intron, or coding site, snpEFF 3.3 [38] was used. Coding sites were further differentiated into synonymous or non-synonymous substitutions based on amino acid changes. Coding regions were also differentiated into transitions and transversions based on changes between or among purines and pyrimidines.

#### Significant QTL and genes

A QTL region is defined as the region around significant stepwise markers that has a partial LD (r^2^) >0.6 with the adjacent marker (corresponds to partial correlation of 0.77). Adjacent blocks were combined if the distance is less than 10 kb and includes up to four markers that are not in LD with either of the block. Candidate genes for iron homeostasis were identified from the research literature, and gene symbols are used from the annotation file available at phytozome (http://www.phytozome.net/soybean.php). A candidate iron gene is considered to be significant in our population if the gene is within the QTL region.

#### Epistatic interactions

To detect epistatic interactions between significant markers, we used a general linear model with both significant markers and their interaction term in the model. We used PC_25_ as covariates in the regression model and an interaction term was considered significant at a p-value of≤1E-03. Genes that are in LD with these significant markers are used to identify the gene-by-gene interactions.

#### Significant allelic combinations

We compared the allelic combinations of the significant markers (0.1 percentile) that were included in stepwise regression. Allelic combinations with a mean IDC<2.5 were termed tolerant, and those with a mean IDC>3.5 were considered susceptible. We chose these values based on standard genotypes grown in the field instead of a statistical approach of mean differentiation, because not all allelic combinations are present in the population. With this approach, the phenotypic distribution does not need to be continuous, where statistically two allelic combinations can be considered significantly different in the absence of intermediate phenotypes.

## Results

### Phenotype and genotyping

**Figure 1:**
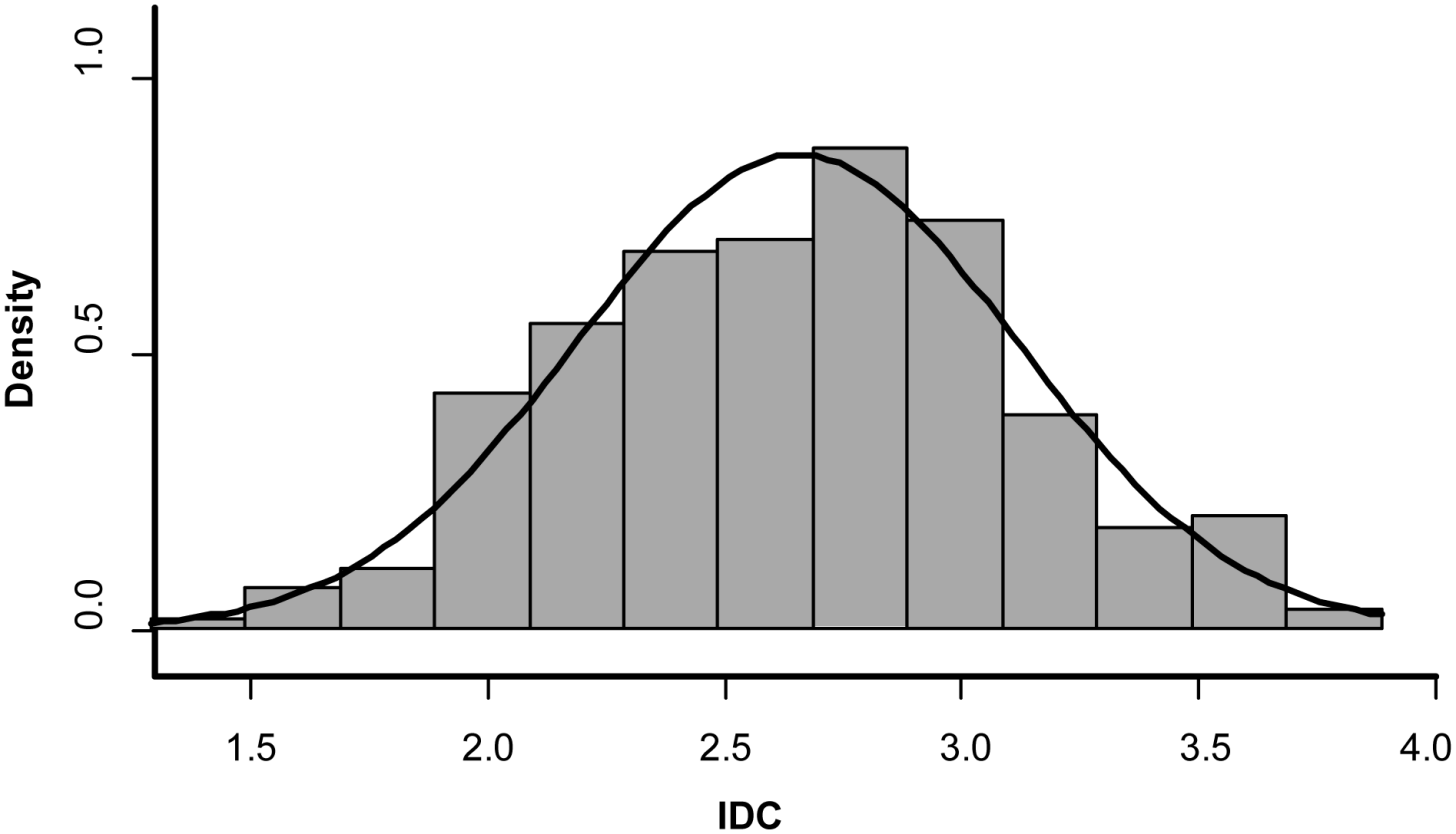
Phenotypic distribution of iron deficiency chlorosis (IDC). The rating of IDC (1–5) is on x-axis and the number of genotypes having the IDC score (represented as density) are presented as y-axis. The curve over the histogram represents the normal distribution of the IDC scores in the population.

For the combined population, the range of IDC is 1.46 to 3.84 with an average of 2.76. IDC scores were normally distributed with a p-value of 0.15 for Kolmogorov–Smirnov test (Figure 1). An average of 1,645,563 reads was obtained for each genotype, and ∼92% of reads mapped to the reference genome. A total of 79,000 putative SNPs that are bi-allelic were identified in the population using the TASSEL-GBS analysis pipeline after removing SNPs that have greater than 50% missing data. The mean coverage depth for each identified SNP was 6.85±2.72.

### Association Mapping

#### Imputation

Missing data (37.5%) was estimated using imputation. Of the 79,000 SNPs, 34,428 had a MAF>0.05 (Table S1). Based on the genome size of *Glycine max*, a polymorphic marker was located, on average, every 31 kbp. In addition, the maximum distance between two adjacent markers did not exceed 500 Kbp providing a good coverage of the SNPs. Of these polymorphic SNPs, 6,520 mapped to a coding region, 6,898 are located in the non-coding region of a gene model and 21,010 are in an intergenic region. The transition to transversions ratio was 1.67.

#### Linkage Disequilibrium Decay

A nonlinear regression model that fits the partial r^2^ of pairwise intra-chromosomal comparisons to physical distance was developed. The average decay of LD (r^2^) in terms of physical distance declined to r^2^ = 0.7 at ∼500 kb (Figure S1). The physical distance at which r^2^ = 0.2 is 3.2 Mbp and at r^2^ = 0.1 was 8.0 Mbp.

#### Population structure, Kinship and model selection

**Figure 2:**
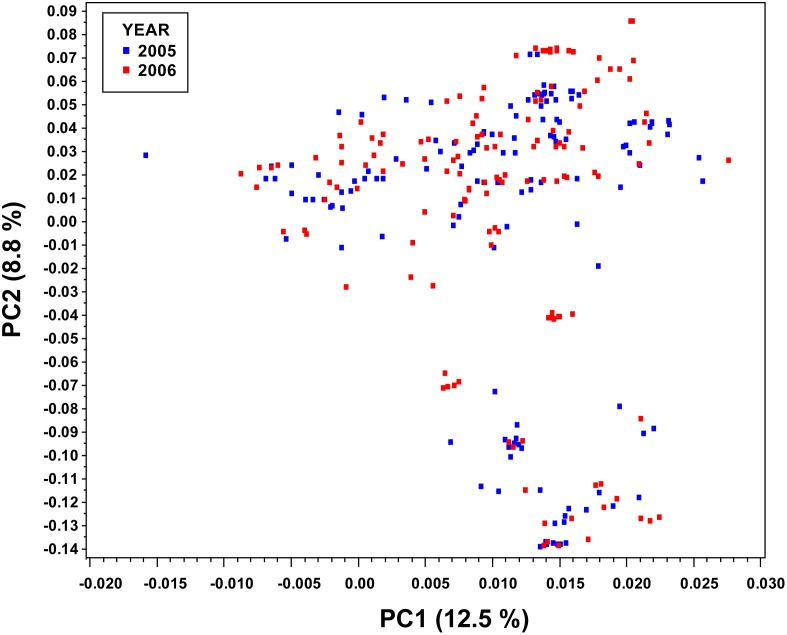
A graph of the first two principal components obtained from all the 34,428 polymorphic SNPs. This graph explains the similarities between the two independent populations and the overall population structure.

**Figure 3:**
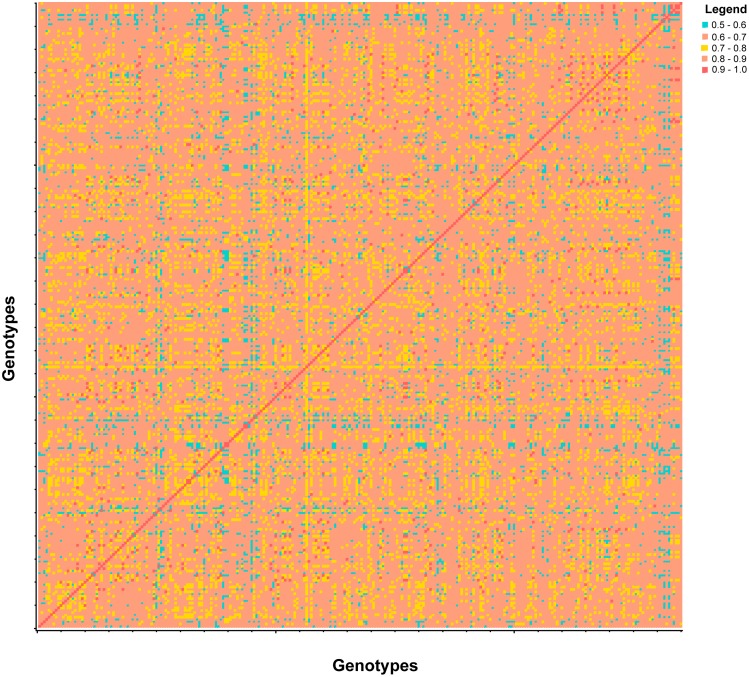
Heatmap of Gower’s similarity distance among the individuals of the population. Based on the colors distribution, it is evident that relatedness can have a major effect on identifying associations to IDC in the population.

**Figure 4:**
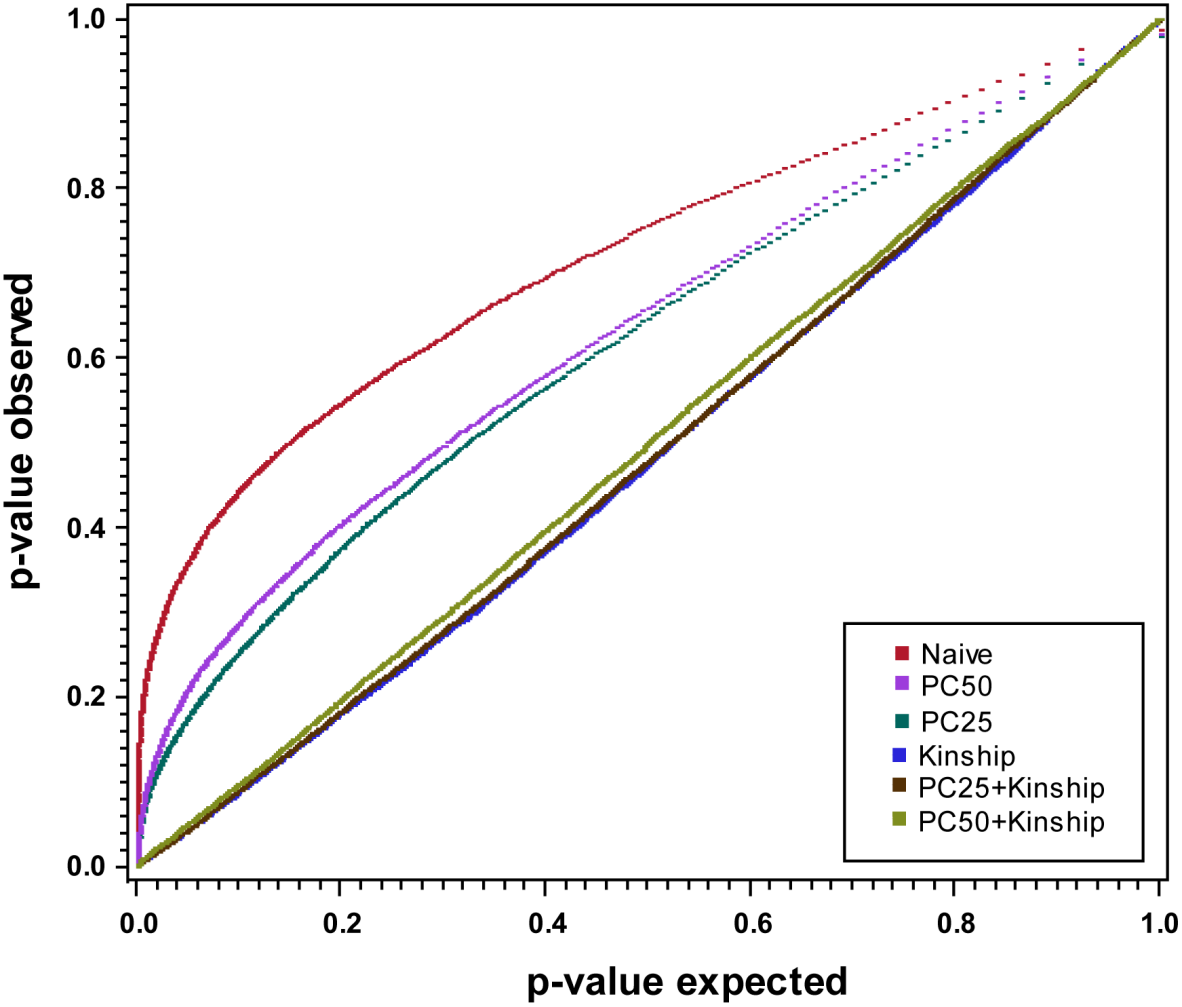
QQ plot for the six models tested. P-value observed is plotted on the y-axis and P-expected is plotted on the x-axis. Each color represents a model of regression used. The best model is the one that is close to the diagonal line.

For the population, three and 13 PC explained 26.8% and 50.5% of the cumulative genotypic variation. Based on the first two PCs, two distinct clusters of populations were observed which indicated the presence of a subpopulation structure (Figure 2). In the population, 80% of the Gowers similarity coefficients are within the range of 0.6 to 0.7 (Figure 3) indicating the presence of relatedness among individuals. This is expected given the narrow genetic base of the breeding population. For the six models tested, the best linear model for the population included 13 PCs (PC_50_) and kinship matrix (MSD = 6.06E-06; Figure 4).

#### Marker Trait associations

**Figure 5:**
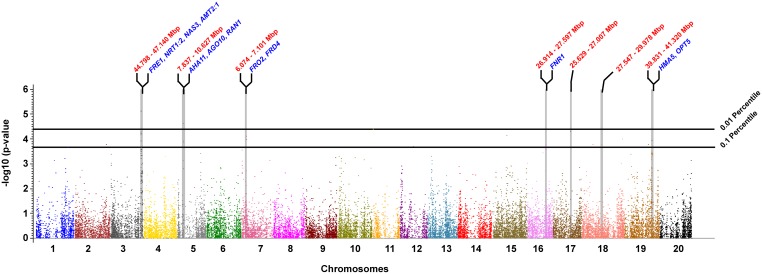
Manhattan plots with significant QTL that are associated with IDC. Chromosomes (1–20) ordered on x-axis and each chromosome is represented by a different color. The –log_10_(p-value) is presented on the y-axis. The cutoff horizontal lines indicate 0.01 and 0.1 percentile tails of the empirical distribution obtained using 10,000 bootstraps. Vertical grey blocks indicate QTL regions that have major effect on IDC trait identified by stepwise regression. The boundaries of the QTL region (based on the position of markers) and significant candidate genes are represented on the top of QTL.

Five markers were significant at the 0.01 percentile level (p≤4.0E-05). One located on Gm03 at 45.03 Mbp, one on Gm05 at 8.82 Mbp and three on Gm11 at 0.53 Mbp (Table 1, Fig. 5). The variation explained by these markers ranged from 5% to 16%, and the mean allelic difference is between 0.31 and 0.38. Stepwise regression included three markers (one from each of the chromosome). Together these three markers explained 23.2% of phenotypic variation.

**Table 1:** Significant markers associated with IDC in the population of soybean.

Chromosome	Position	–log10 (p-value)	Reference Allele	Variant Allele	R^2^ §	MAF§§	Mean Difference	Additive Effect (Variant allele)	Included in stepwise regression§§§
Gm02	46,939,135	3.784**	A	T	5.20	21.48	0.26	−0.13	
Gm03	44,927,455	4.165**	A	G	15.20	38.89	0.37	−0.19	
Gm03	44,930,187	4.165**	A	T	15.20	38.89	0.37	−0.19	
Gm03	44,970,860	4.275**	A	G	14.42	39.26	0.36	−0.18	
Gm03	44,985,022	4.165**	G	A	15.20	38.89	0.37	−0.19	
Gm03	45,031,929	5.127***	T	C	15.77	38.89	0.38	−0.19	x
Gm03	45,038,912	3.684**	C	T	14.71	39.26	0.36	−0.18	
Gm03	45,107,141	3.905**	C	A	13.87	45.56	0.35	−0.17	
Gm03	45,343,317	3.689**	G	T	17.28	42.59	0.39	−0.19	
Gm03	45,343,612	4.231**	A	T	18.85	41.48	0.41	−0.20	
Gm05	8,817,714	3.912**	G	A	10.81	49.26	0.30	0.15	
Gm05	8,823,135	4.617***	G	A	11.43	49.63	0.31	0.16	
Gm05	8,877,264	3.855**	G	T	8.30	45.93	0.27	0.13	x
Gm05	17,397,118	3.704**	G	A	0.08	39.63	0.03	0.01	
Gm05	17,397,130	3.704**	T	C	0.08	39.63	0.03	0.01	
Gm05	17,397,135	3.704**	C	T	0.08	39.63	0.03	0.01	
Gm07	6,397,319	3.961**	C	T	8.80	25.56	0.32	−0.16	x
Gm07	6,405,421	3.961**	T	C	8.80	25.56	0.32	−0.16	
Gm07	6,540,541	4.128**	G	A	8.10	26.30	0.30	−0.15	
Gm07	6,575,306	3.961**	A	G	8.80	25.56	0.32	−0.16	
Gm11	530,116	4.401***	A	G	4.73	8.89	0.35	0.18	x
Gm11	530,117	4.401***	A	C	4.73	8.89	0.35	0.18	
Gm11	530,118	4.401***	G	T	4.73	8.89	0.35	0.18	
Gm12	20,406,669	3.697**	T	C	0.02	20.74	0.01	0.01	
Gm15	21,422,057	4.152**	A	G	2.10	20.37	0.17	0.08	
Gm16	27,300,116	3.779**	T	G	4.80	8.52	0.36	0.18	x
Gm17	25,859,992	4.322**	T	C	0.66	6.67	0.15	0.08	x
Gm17	25,859,995	4.322**	T	C	0.66	6.67	0.15	0.08	
Gm18	15,905,372	3.781**	T	C	3.25	28.52	0.18	0.09	
Gm18	28,141,888	3.786**	G	A	3.78	22.59	0.22	0.11	x
Gm18	59,284,729	4.006**	C	T	0.70	45.93	0.08	0.04	
Gm19	34,914,546	3.769**	A	G	0.81	5.19	0.19	0.09	
Gm19	40,193,564	3.955**	C	A	15.62	44.07	0.37	−0.18	x

***Significant at 0.01 percentile (4.0E-05).

**Significant at 0.1 percentile (2.1E-04).

§ R^2^– Calculated using a simple linear regression and is represented as percentage.

§§ MAF = Minor Allele Frequency.

§§§ The markers that have an ‘x’ are included in stepwise regression. All other significant markers are in LD with one of these eight markers. This is the subset of markers around which QTLs were identified, and allelic combinations that have applications for MAS are characterized.

At a significance level of 0.1 percentile (p≤2.12E-04), 33 significant markers were distributed on 10 of the 20 chromosomes (Table 1, Figure 5). Eight of these markers included in stepwise regression are on Gm03 (45.03 Mbp), Gm05 (8.87 Mbp), Gm07 (6.39 Mbp), Gm11 (0.53 Mbp), Gm16 (27.30 Mbp), Gm17 (25.85 Mbp), Gm18 (28.14 Mbp), and Gm19 (40.19 Mbp). These markers together explained 46.3% variation.

#### Annotation of SNP

Of the significant markers at 0.1 percentile, five SNP are present in a coding region (three are transitions and two are transversions), 11 in introns, two in 3′UTR regions, and 15 in intergenic regions. Of the five SNPs from a coding region, two are non-synonymous substitutions. For FRE1 (Glyma03G38620), one of these amino acid changes is associated with both a polarity and a charge change of the protein. This SNP at position 44,927,455 bp of Gm03 changes an asparagine to aspartic acid. The R-square explained by this marker alone is 15.2% for the population. The variant allele ‘A’ is more tolerant than reference allele. The other non-synonymous substitution is located in the gene model (Glyma03g39151) close to the above non-synonymous substitution at 45,343,612 bp. This SNP explains about 18.85% of the phenotypic variation in the population and has a partial r^2^ of 0.80 with the above marker. These two markers together explain 19.0% of phenotypic variation.

#### Significant QTL and genes

**Figure 6:**
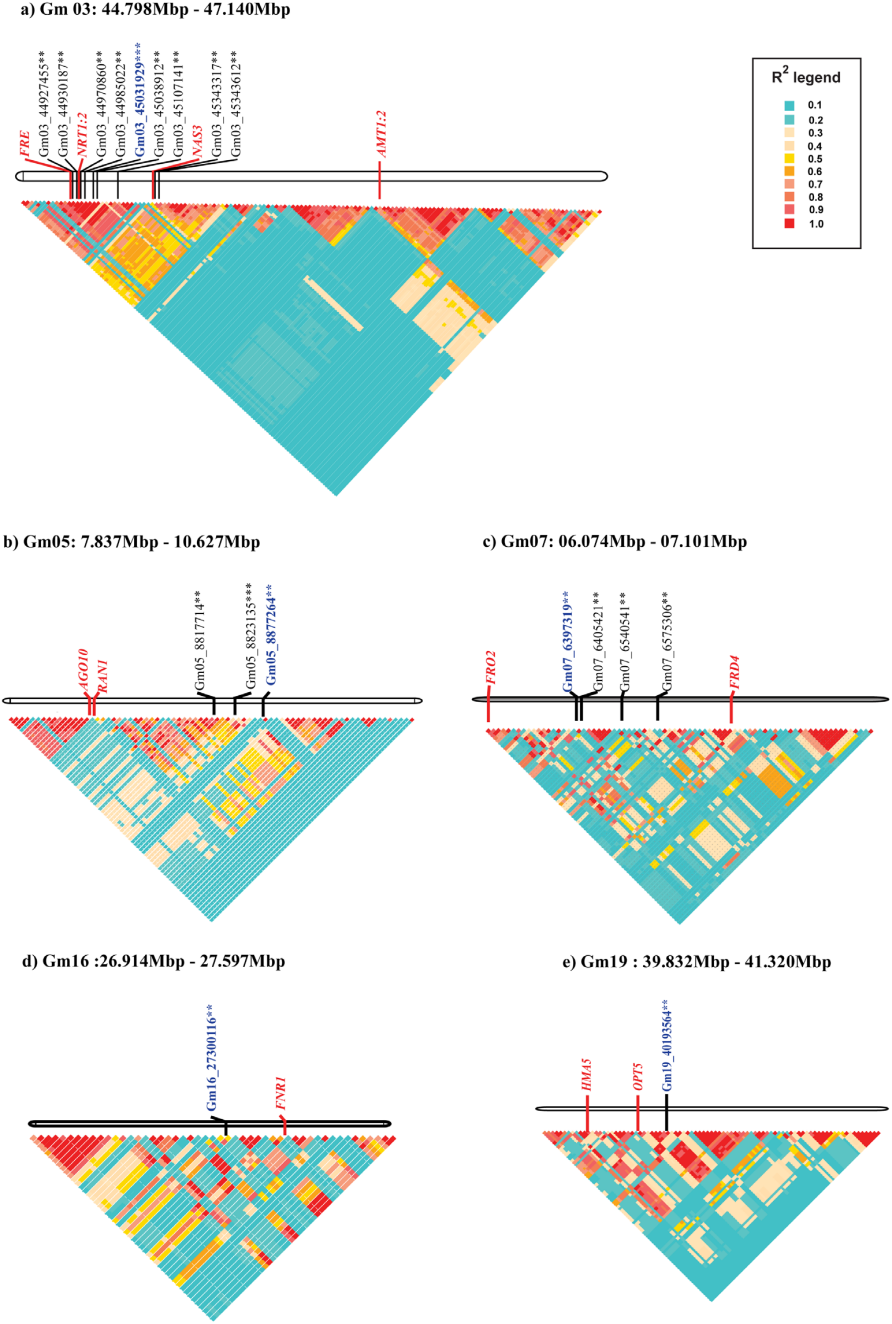
Heatmap of major QTL regions. QTL that has known candidate gene are only represented. Only significant markers at the 0.1 percentile bootstrapped empirical distribution were included in the figure. Significance of the markers is indicated as *** if fall in the 0.01 percentile tail and ** if fall in the 0.1 percentile tail of the bootstrapped empirical distribution. The stepwise included markers are marked in blue. r^2^ values are represented in different colors. Marker pairs with r^2^ values>0.6 are considered to be in linkage disequilibrium. a) is the QTL on Gm03 (44.798 Mbp–47.140 Mbp), b) is the QTL on Gm05 (7.837 Mbp–10.627 Mbp), c) is the QTL on Gm07 (6.074 Mbp–7.101 Mbp), d) is the QTL on Gm16 (26.914 Mbp–27.597 Mbp) and e) is QTL region on Gm19 (39.832 Mbp–41.320 Mbp).

At the significance level of 0.01 percentile, three markers were included in the stepwise regression. One of these marker is in LD (r^2^≥0.6) with the adjacent markers, and the QTL is located on Gm03 between 44.798 Mbp and 47.140 Mbp (Table 2; Figure 5; Figure 6). The two major candidate genes in this QTL are phytochrome A (FRE1; Glyma03g38620) and nicotianamine synthase 3 (NAS3; Glyma03g39050). In addition to these genes, nitrate transporter 1∶2 (NRT1∶2; Glyma03g38640) and ammonium transporter 2 (AMT2; Glyma03g40700), each with a role in iron homeostasis, are also located within the QTL region. The QTL around the significant marker on Gm05 is between 7.837 and 10.627 Mbp (Table 2; Figure 5; Figure 6). The candidate genes in this QTL include stabilizer of iron transporter (AGO10, PNH, ZLL; Glyma05g08170), RAS-related nuclear protein-1 (RAN1; Glyma05g08260) and H (+)-ATPase 11 (AHA11; Glyma05g01460). The third significant marker on Gm11 is located in the 5′ UTR region of the CVP2 like 1 gene (CVL1; Glyma11g00990). The marker is not in LD with any of the adjacent markers.

**Table 2:** Significant QTL regions associated with IDC that have Candidate iron genes.

Gene Model*	Chrom	Start	End	Genesymbol*	Annotation*	Function
**Gm03: 44.798–47.140**						
Glyma03g38620	Gm03	44,925,730	44,930,360	FRE1	Phytochrome A	Increased reductase activity and better tolerance in high pH soils [47]–[48].
Glyma03g38640	Gm03	44,943,759	44,948,193	NRT1∶2	Nitrate Transporter 1∶2	Increase concentration of nitrate in plants and lead to increased growth and root extension [71]–[72].
Glyma03g39050	Gm03	45,279,743	45,281,178	NAS3	Nicotianamine Synthase 3	Synthesis of iron chelator, nicotiamine [67]–[68].
Glyma03g40700	Gm03	46,372,699	46,374,428	AMT2;1	Ammonium Transporter 2	Increase concentration of nitrate in plants and lead to increased growth and root extension [71]–[72].
**Gm05: 7.837–10.627**						
Glyma05g01460	Gm05	960,779	967,307	AHA11	H(+)-ATPase 11	Limits transport of iron in plants [60].
Glyma05g08170	Gm05	8,130,422	8,137,850	AGO10	Stabilizer of iron transporter SufD/Polynucleotidyl transferase	Stress response [82].
Glyma05g08260	Gm05	8,219,986	8,222,377	RAN1	RAS-related nuclear protein-1	Involved in iron transport [76].
**Gm07: 6.074–7.101**						
Glyma07g07380	Gm07	6,059,979	6,064,461	FRO2	Ferric reduction oxidase 2	Reduce iron at cell surface before transport [61]
Glyma07g08240	Gm07	6,837,072	6,844,618	FRD4	Signal recognition particle receptor protein, chloroplast (FTSY)	Reduces iron in response to iron deficiency [56].
**Gm16: 26.914–27.597**						
Glyma16g23710	Gm16	27,560,086	27,563,676	FNR1	Ferrodoxin NADP (+) oxidoreductase 1	Decreased chlorophyll biosynthesis [64].
**Gm19: 39.831–41.320**						
Glyma19g32190	Gm19	39,950,268	39,955,547	HMA5	Heavy Metal Atpase 5	Maintain metal balance under Fe deficiency [78]–[80].
Glyma19g32401	Gm19	40,141,923	40,144,157	OPT5	Oligopeptide transporter 5	Oligopeptide transporter [77].

*Gene Model, gene symbol and annotation obtained from phytozome (www.phytozome.net/soybean, V8.0).

At a significance level of 0.1 percentile, eight markers are included in the stepwise regression model. These eight markers are located in eight QTL regions. The QTL on Gm03, Gm05 and Gm11 are the same as the ones identified at the 0.01 percentile level. The other QTL at this significance level include Gm07 (6.074 Mbp–7.101 Mbp), Gm16 (26.914–27.597 Mbp), Gm17 (25.629–27.007 Mbp), Gm18 (27.547–29.978 Mbp), and Gm19 (39.831–41.320 Mbp) (Table 2; Figure 5; Figure 6). On Gm07, the significant candidate genes include ferric reduction oxidase 2 (FRO2; Glyma07g07380), and signal recognition particle receptor protein on chloroplast (FRD4; Glyma07g08240). On Gm16, the significant candidate gene ferrodoxin NADP (+) oxidoreductase 1 (FNR1, Glyma16g23710) is within the QTL region. On Gm19, heavy metal atpase-5 (HMA5; Glyma19g32190), and oligopeptide transporter-5 (OPT5; Glyma19g32401) are significant.

Other than the genes involved in iron metabolism, several genes involved in disease resistance are also identified in the QTL regions. For example, QTL on Gm03 has two RING/U-box superfamily proteins that have a role in plant defense. Similarly on Gm05, two CC-NBS-LRR, one NBS and one LRR genes are present. On Gm07 within the QTL region, three NBS genes are present. On Gm16, seven LRR proteins, and on Gm19, six NBS proteins, two LRR and one RING/U-box superfamily proteins are present.

#### Epistatic interactions

With 33 significant markers at 0.1 percentile, 528 two way interactions were tested with a population structure component, and five interactions were significant at p-value<0.001. Three of these interactions are between Gm07 (around 6.4 Mbp) and Gm18 (59.28 Mbp). The other significant interactions are between Gm16 (27.30 Mbp) and Gm18 (59.28 Mbp), Gm02 (46.939) and Gm15 (21.42 Mbp). The genes within LD regions include cytochrome P450 like protein (Glyma18g50051) on Gm18 with FRO2 (Glyma07g07380) on Gm07 and FNR1 (Glyma16g23710) on Gm16. The genes on Gm02 include AUX1 (Glyma02g42290) and FER4 (Glyma02g43040) which interact with CCC1 (Glyma15g22520) on Gm15.

#### Significant Marker Allelic combinations

Allelic combinations of SNPs can be used to infer the tolerant/susceptible phenotype of a genotype. For the significant markers (0.1 percentile) that were included in stepwise regression in the population, 14 allelic combinations are predicted as tolerant (IDC mean≤2.5) and 6 allelic combinations indicate susceptibility (IDC mean≥3.5) (Table 3).

**Table 3:** Allelic combinations and IDC means for the allele combinations that have a mean<2.5 (Tolerant) and>3.5 (Susceptible).

Allelic combination§	# of genotypes	IDC			
		Minimum	Maximum	Mean	Standard Deviation
CTTATTGA	3	1.63	2.23	1.88	0.31
TGTATTGA	7	1.46	2.58	2.17	0.37
CGTAGTGA	2	2.18	2.34	2.26	0.11
CTTATTGC	6	1.96	2.86	2.29	0.33
TTTATTGA	11	1.95	3.25	2.32	0.40
CGCATTGA	9	2.08	2.83	2.38	0.26
CTCGTTAA	1	2.4	2.4	2.40	
CTTATTAA	2	2.16	2.66	2.41	0.35
CTCATTGA	26	1.8	3.01	2.41	0.33
CTTGTTGC	2	2.16	2.69	2.43	0.37
CTCATTGC	9	2.11	3.03	2.44	0.33
CGTATTGA	5	2.31	2.57	2.47	0.13
CTTATCGC	2	2.42	2.52	2.47	0.07
CGTATTGC	1	2.5	2.5	2.50	
TTCGTTAC	1	3.56	3.56	3.56	
TTCAGTAC	1	3.58	3.58	3.58	
TGCAGCGC	1	3.68	3.68	3.68	
TGCGTTGC	1	3.7	3.7	3.70	
TGCGTTAA	1	3.72	3.72	3.72	
TGCGTTAC	1	3.79	3.79	3.79	

§ The order of the markers for the allelic combination are Gm03_45031929, Gm05_8877264, Gm07_6397319, Gm11_530116, Gm16_27300116, Gm17_25859992, Gm18_28141888, and Gm19_40193564.

## Discussion

A higher marker density facilitates identification of high resolution QTL and gene discovery [39], [40]. One method of generating a high frequency of SNPs is next generation sequencing [41], [42]. Using GBS, we identified ∼35,000 polymorphic markers with a minor allele frequency>0.05. This provided good coverage, one marker at every 31 kb. This density is useful for characterizing most genetic factors responsible for soybean IDC given that LD decayed here to r^2^ = 0.7 at 500 kbp.

LD decay assesses the depth of markers required for GWAS. Partial LD was calculated using population structure as a cofactor, and as expected we observed a variation in LD decay when including this as a cofactor. For example, LD decay for the population is at 10 Mbp at r^2^ = 0.1 whereas adjustments for confounding effect of population structure reduced LD decay to 7 Mbp. The use of partial LD (r^2^) helps overcome the bias between adjacent markers and leads to smaller QTL regions.

The population consisted of breeding lines developed by public and private programs targeting the north central states of the US. The germplasm is expected to be narrow given the limited range of maturity group (0–1), the short history of soybean breeding in USA, and the limited number of traits selected in the breeding programs. Here we used multiple mixed models [31], [34], [43]–[45] that control for confounding effect of population structure and population relatedness [16], [46].

The percentile p-value cutoffs derived from empirical distribution were all within the p = 0.001 error level, and the significant markers were distributed on eleven of the twenty chromosomes. This is expected given the complex nature of iron homeostasis in plants. At a significance level of one percentile (p≤2.12E-04), many more markers were significant (345 markers) and explained the majority of the phenotypic variation in the population (R^2^ = 85%). However, owing to the complexity of the trait, we limited the interpretations to the 0.1 percentile to find the major QTL effects.

To determine if any significant SNP is a potential causal variant, we searched for non-synonymous substitutions. One was discovered in Far red elongated 1 (FRE1) which encodes a protein that increase reductase activity and provides a higher level of tolerance in high pH calcareous soils [47]–[49]. The other non-synonymous substitution was discovered in a gene with no known function related to iron homeostasis. This can be expected because the functions of many genes are still unknown [50]–[52].

Stepwise regression facilitates the selection of markers that have a major effect in a QTL region and simultaneously masks the effects of other minor QTL. As an example, the QTL regions on Gm02 and Gm15 that have a significant interaction were not included in the stepwise regression given these QTL have a minor effect. Additionally, the partial LD between the 33 significant markers is 0.09 (±0.247). But for the eight markers included in stepwise regression, partial LD (r^2^) is 0.008 (±0.021). This subset of markers included in stepwise regression explains 23.2% and 46.3% of the 0.01 and 0.1 percentile, respectively. This is similar to the variation explained by all significant markers (23.2% and 48.6% at the 0.01 and 0.1 percentile, respectively).

Comparing with independent populations, the combined population analysis was able to identify markers from eight addition QTL regions that are not discovered in independent populations at significance level of 0.1 percentile. Only six QTL regions are common with either one of the population. Further using a stepwise regression to identify major effect QTL, the combined analysis was able to identify three unique QTL regions (On Gm05, Gm17, and Gm18) that were not identified in the independent populations. Of the other five QTL regions, two are common with 2005 population (on Gm03, Gm11) and three are common with 2006 population (Gm07, Gm16, Gm19).

For the population, we identified seven QTL on seven chromosomes. Given the complex quantitative nature of iron metabolism, multiple QTL distributed throughout the genome are expected. The region on Gm03 is in the same region identified earlier by Severin et al. [14] and Stec et al. [15] using an introgression analysis and is a significant QTL in many biparental studies [53]–[55]. In addition to this major QTL, other QTL regions were also identified earlier. For example, QTL region on Gm05 were identified earlier by Severin et al. [14]. The QTL on Gm16 is close to an introgression region identified earlier [14], [15]. Similarly Lin et al. [53] identified QTL on Gm18 and Gm19 and Lin et al.[56] identified QTL on Gm18. Most of these regions were also identified earlier using the same population with about 1000 polymorphic markers [9]. The major difference of this study from the previous one is the ability to narrow the QTL region and the ability to study the entire genome given a polymorphic marker every 31 kb. In addition we were able to identify significant SNP markers within the candidate genes.

Iron is a structural component of many important processes in plants, and multiple physiological steps are used by strategy-I plants, such as soybean, to manage plant growth and development in iron-deficient conditions [56]. Protons release results in the acidification of the rhizosphere which increases the solubility of iron. Ferric iron is reduced at the root surface, and ferrous iron uptake increases [57]–[59]. Here we discovered multiple genes located in major QTL associated with these major activities.

AHA11 (Gm05) is an abundant protein found in the seedlings and leaves [60] and limits the transport of iron to leaves resulting in chlorotic plants. Ferric-chelate reductase oxidase (FRO2; Gm07) is expressed in the epidermal cells of Fe-deficient roots and is one of the main Fe (III) chelate reductase proteins. It reduces iron at the cell surface before transport into roots [61]. FRO2 also interacts with IRT1 under Fe sufficiency conditions to increase iron uptake [62], [63]. FRE1 (Gm03) increases reductase activity and tolerance to high pH in calcareous soils [47], [48]. Based on transgenic lines in *Oryza*, Ishimaru et al. [47], [48] suggested that this gene is regulated by the IRT1 since transgenic lines have a higher level of Fe uptake and enhanced tolerance to low Fe availability in both hydroponic culture and calcareous soils.

Ferric chelate reductase defective (FRD4; Gm07) is a gene that acts post-translationally and reduces Fe (III) chelate reductase activity in response to iron deficiency [56]. In addition, *frd4* mutants are chlorotic, grow more slowly, have smaller chloroplasts, and possess fewer thylakoid membranes and grana stacks. Map-based cloning revealed that this gene encodes a component that is responsible for the insertion of proteins into the thylakoid membranes of the chloroplast [56]. Ferredoxin NADP reductase 1 (FNR1; Gm16) is an enzyme that catalyzes reversible electron transfer in photosynthetic electron transport. In iron stress conditions, its activity is decreased resulting in reduced chlorophyll biosynthesis [64].

Nicotianamine (NA) is an iron chelator involved in iron (Fe) acquisition, transport, and homeostasis [65], [66], and NA is synthesized by nicotianamine synthase that is encoded by *NAS3* (Gm03) gene. Overexpression of NAS genes in rice increased Fe and Zn uptake by at least twofold [67], [68]. Nicotianamine synthase genes are induced by nitrate levels [69], [70]. Ammonium and nitrate transporters (Gm03) increase the uptake of nitrate from rhizosphere and increase the concentration of nitrate in plants and lead to increased growth and extension of roots [71], [72]. In addition, nitrates enhance H+ extrusion and acidify the rhizosphere leading to increased bioavailability of Fe [71]–[75].

Several transporters such as RAN1 (Gm05), OPT5 (Gm19), and HMA (Gm19) are in the significant QTL regions. RAN1 is involved in copper trafficking and iron transport [76]. OPT5, an oligopeptide transporter is highly expressed in multiple organs of adult plants [77], and HMA sequesters Cd, Zn, and Ni in vacuoles under Fe deficiency to maintain metal balance and to detoxify heavy metals [78]–[80].

Argonaute (AGO) like proteins are down-regulated under conditions of low Fe and phosphorus [81]. Vaucheret [82] suggested that these genes have functions in both development and stress response by regulating gene expression at various levels and has additional functions including chromatin remodeling, DNA methylation, translational repression, and RNA cleavage. One such *AGO* gene, *AGO10* (Gm05) is in a major QTL.

Disease resistant genes (R genes) are distributed throughout the *G.max* genome and are a key component of gene interactions between plants and pathogens [83]. We identified multiple R genes in IDC QTL regions. Iron is an important element for all living organisms and pathogens and hosts actively compete for available metals. Plants need metals for defensive generation of reactive oxygen species (ROS) and other plant defenses that limit pathogen growth whereas pathogens use low-metal conditions as a signal to recognize and respond to the host environment [84]. Pathogens have developed sophisticated strategies to acquire metal during plant growth that include production of multiple siderophores. Chen et al. [85] studied the interaction IDC and soybean cyst nematode (SCN) resistance and discovered SCN susceptible varieties had higher IDC rating. Since there exists a significant interaction between diseases and IDC, selecting for disease resistance may improve IDC tolerance.

For many traits, estimating epistatic interactions in plants [86], [87] has been restricted to bi-parental populations [50], [88]–[90]. For the population, FRO2, a proton with reductase activity interacts with Cytochrome P450 like proteins. Cytochrome P450 proteins accumulate in response to iron deficiency [91]. If iron uptake is limited by the function of FRO2, it would lead to iron deficiency and limit AUX1 and initiate cytochrome P450 activities. Similar to Cytochrome P450 proteins, activity of FNR is reduced under iron deficient conditions [64]. AUX1 is a gene that is involved in auxin transport, a plant hormone that accumulates in Fe-deficient Arabidopsis roots and acts upstream of nitric oxide [92]. FER4 is a gene responsible for cellular iron homeostasis and subcellular iron trafficking [93]. CCC is a transporter molecule that transports iron [94]. If the activity of the transporter is reduced, auxin accumulates more and the activity of FER4 is reduced.

Evaluating allelic combinations of markers can define appropriate groups of markers for MAS. It is well understood, that there is no single universal marker that is significant in all populations. This is because of the altered effect of the genes and the QTL. It is the combination of these markers that have a significant difference on the phenotype. Of the many allelic combinations possible for multiple SNP (2^n^, where n is the number of biallelic markers), only a few of them represent the extremes of phenotype. We provide allelic combination details for the extreme phenotypes for the significant markers that are included in the stepwise regression.

## Conclusion

We used a high density GBS data set with a large plant GWAS population to map genes/QTL involved in IDC of soybean. Stepwise regression was used to select a subset of markers that have a major effect on IDC. We identified several significant QTL and candidate iron genes confirming the polygenic nature of the trait. Among these, a non-synonymous substitution identifies FRE1 as a candidate gene. Stepwise regression was effective for selecting the subset of markers that have major effect. This subset of markers can further be used for validation and testing in other populations. A high throughput study on expression differences of genes and the variants responsible for these differences would further help breeders identify varieties tolerant to IDC.

## Supporting Information

Figure S1 **Genome-wide linkage disequilibrium (LD) decay plot for the population.** Linkage disequilibrium, measured as partial R^2^, between pairs of polymorphic marker loci (intra-chromosomal comparisons) is plotted against the physical distance (Mbp).
(PDF)

Table S1 **Detailed information of SNPs used in the study.** SNP postions, alleles based on soybean reference genome V1.1 (phytozome.org) and minor allele frequency for this population (in %).
(XLSX)
