# Supplementary material for: Genome-Wide Association Studies Identifies Seven Major Regions Responsible for Iron Deficiency Chlorosis in Soybean (Glycine max)
Source: PLoS One. 2014 Sep 16;9(9):e107469. doi: 10.1371/journal.pone.0107469 (PMC4166409; doi:10.1371/journal.pone.0107469)
Supplement: Figure S1 — Genome-wide linkage disequilibrium (LD) decay plot for the population. Linkage disequilibrium, measured as partial R2, between pairs of polymorphic marker loci (intra-chromosomal comparisons) is plotted against the physical distance (Mbp). (PDF) [file pone.0107469.s001.pdf]

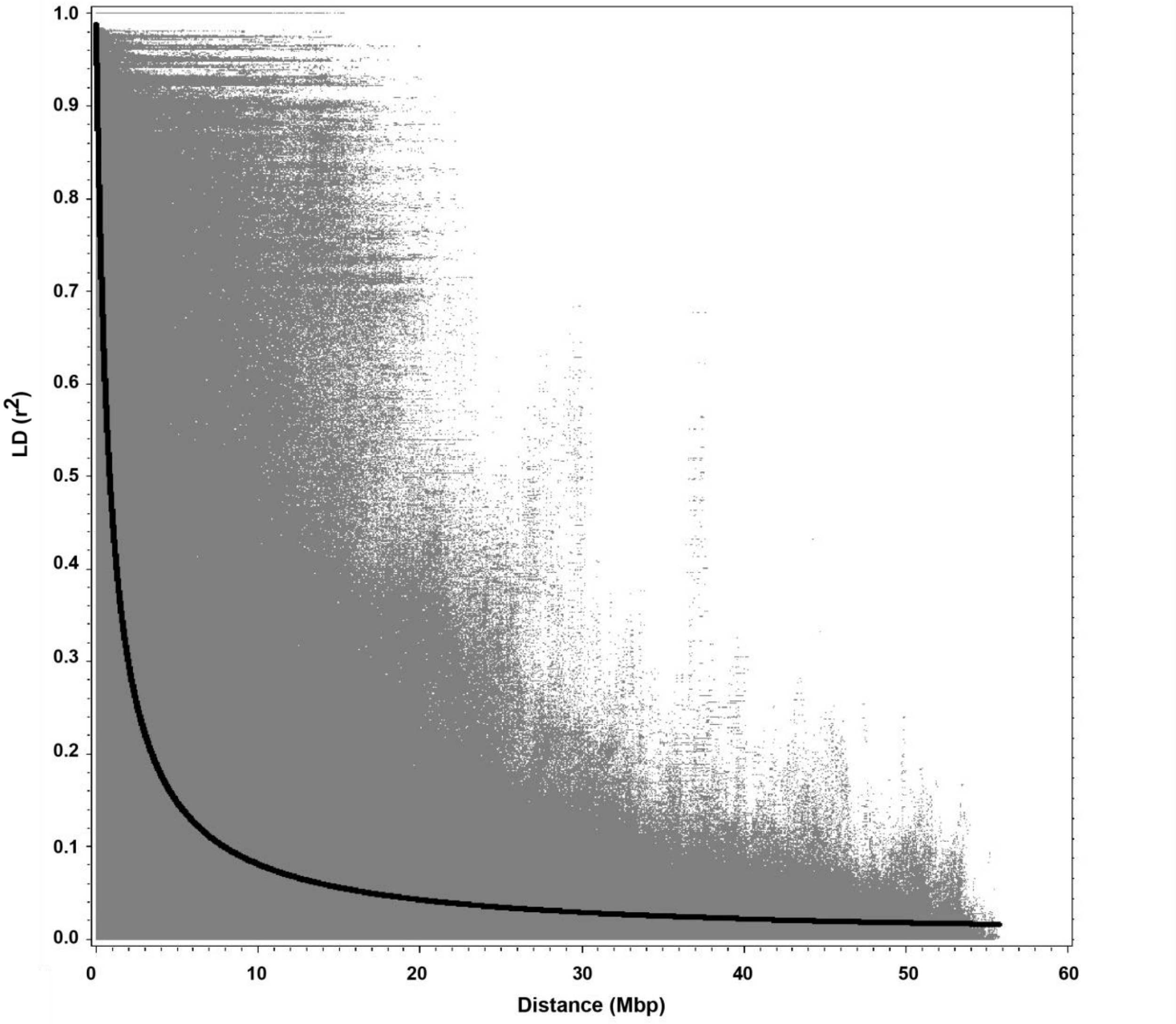

Supplementary Figure 1: Genome-wide linkage disequilibrium (LD) decay plot for the population. Linkage disequilibrium, measured as R^2, between pairs of polymorphic marker loci (intra-chromosomal comparisons) is plotted against the physical distance (Mbp).
